# Romosozumab: a novel bone anabolic treatment option for osteoporosis?

**DOI:** 10.1007/s10354-019-00721-5

**Published:** 2019-12-19

**Authors:** Katharina Kerschan-Schindl

**Affiliations:** Department of Physical Medicine, Rehabilitation and Occupational Medicine, Medical University of Vienna, Waehringer Guertel 18–20, Vienna, 1090 Austria

**Keywords:** Sclerostin, Romosozumab, Blosozumab, Side effects, Sclerostin, Romosozumab, Blosozumab, Nebenwirkungen

## Abstract

Research into the drug romosozumab began with the investigation of patients with excess bone formation. The understanding of the wingless-type mouse mammary tumor virus integration site (Wnt) signaling pathway in bone metabolism identified the negative regulator of bone mass sclerostin as a potential target for the treatment of osteoporosis. Preclinical studies confirmed this idea because they showed that sclerostin antibodies have the potential to increase bone formation. Biochemical analyses of clinical studies showed a significant increase in bone formation markers, which then slowly decreased within a year. This was accompanied by a particularly initially pronounced decrease in bone resorption. This dual mechanism of action led to an increase in bone mineral density and a significant reduction in fracture risk. Clinical vertebral fractures decreased by between 28 and 36%, nonvertebral fractures shown in a post hoc analysis by 42%. Romosozumab is administered once a month in the form of two injections. At the puncture site, reactions occur in about 5%. The most significant side effects are cardiovascular. In phase III studies, the number of serious cardiovascular complications was not significantly, albeit numerically, higher than in the control group. In Japan, South Korea, Canada, Australia, and the USA, osteoporosis patients at a high risk of fracture may already be treated with romosozumab (Evenity). Approval in the European Union was granted by 2019-12-12.

## Zusammenfassung

Die Erforschung des Medikaments Romosozumab nahm ihren Anfang in der Untersuchung von Personen mit übermäßiger Knochenbildung. Mit dem Verständnis des Wnt(„wingless-type mouse mammary tumor virus integration site“)-Signalwegs wurde Sclerostin, ein negativer Regulator der Knochenmasse, als potenzieller Angriffspunkt in der Osteoporosetherapie identifiziert. Präklinische Studien bestätigten dieses Konzept, denn sie zeigten, dass durch Sclerostin-Antikörper die Knochenbildung gesteigert werden kann. Biochemische Analysen klinischer Studien ergaben einen deutlichen Anstieg der Marker für die Knochenbildung, welche dann langsam innerhalb eines Jahres abfielen. Dies wurde begleitet von einem anfangs besonders stark ausgeprägten Abfall der Knochenresorption. Dieser duale Wirkmechanismus führte zu einem Anstieg der Knochenmineraldichte und einer signifikanten Reduktion des Frakturrisikos. Klinische vertebrale Frakturen nahmen zwischen 28 und 36 % ab, nichtvertebrale Frakturen gezeigt in einer post hoc Analyse um 42 %. Die Gabe von Romosozumab erfolgt einmal monatlich in Form von 2 Injektionen. An der Einstichstelle kommt es in etwa 5 % zu Reaktionen. Die bedeutendsten Nebenwirkungen finden sich im kardiovaskulären Bereich. In den Phase-III-Studien war die Anzahl der schwerwiegenden kardiovaskulären Komplikationen nicht signifikant, aber numerisch höher als in der Kontrollgruppe. In Japan, Südkorea, Kanada, Australien und den USA können Osteoporosepatienten mit hohem Frakturrisiko bereits mit Romosozumab (z. B. Evenity) behandelt werden. In der Europäischen Union ist das Präparat seit dem 12.12.2019 zugelassen.

In healthy adults, bone resorption and bone formation are coupled. The process of bone remodeling is essential for the maintenance of bone mass. With increasing age, bone loss—a deficit of bone formation relative to bone resorption [1]—occurs. The insufficiency of osteoblast activity is caused by molecular mechanisms.

Essential for osteoblast differentiation and activity is the wingless-type mouse mammary tumor virus integration site (Wnt) pathway. Activation of the canonical anabolic Wnt/ß-catenin pathway occurs by binding of Wnt proteins to the extracellular part of the receptor complex consisting of frizzled (FRZ) and lipoprotein receptor-related proteins 5 and 6 (LRP‑5 and LRP-6). Generated signals inhibit the activity of glycogen synthase kinase 3 (GSK 3) and destroy the ß‑catenin destruction complex. Stabilized ß‑catenin translocates into the nucleus and induces the transcription of osteoblastic proteins [2]. Wnt proteins, co-receptors, intracellular molecules, and transcription factors tightly regulate Wnt signaling [2]. Well-known modulators and important inhibitors of this canonical Wnt pathway are dickkopf 1 (Dkk 1) and sclerostin [3, 4].

## Sclerostin

Sclerostin was first recognized when diseases associated with high bone mass caused by mutations of the *SOST* gene were studied. Defects in the *SOST* gene were described as early as in the 1950s, [5, 6]: Van Buchem disease or “hyperostosis corticalis generalisata familiaris” is caused by deletion of an element of the *SOST* gene. Sclerosteosis, which is mainly found in South Africa, is the result of a homozygous mutation in the *SOST* gene. In both diseases, loss of function of the negative regulator of bone formation sclerostin leads to abnormal formation of bone. Due to narrowing of the cranial nerves’ foramina, clinical symptoms like facial palsy, hearing impairments, or raised intracranial pressure occur [7, 8]. In sclerosteosis, the more severe disease, patients may also suffer from syndactyly.

Sclerostin, the product of the *SOST *gene, is a glycoprotein consisting of about 200 amino acids. Sclerostin mRNA has also been found in chondrocytes, kidney, lung, vasculature, and heart [9]. However, sclerostin is supposed to be mainly produced by bone matrix-embedded osteocytes. The mode of action of this Wnt antagonist is binding to and thus inactivating LRP [10]. Consequently, osteoblast differentiation and activity are reduced.

Despite the local action of sclerostin, biochemical analyses of circulating sclerostin seem to give a good impression of sclerostin levels in bone [11, 12]. Serum sclerostin levels are regulated by physiologic and pathophysiologic conditions. Although the expression of sclerostin was not altered in aged osteoblasts in an in vitro study [13], all clinical studies [14–17], except for one which also included subjects with chronic diseases like diabetes mellitus [18], found a positive correlation with age. In men as well as in women, the age-associated increase of serum sclerostin levels may be induced by the age-associated decline of estrogen [14]. Two studies detected higher serum sclerostin levels in men than in women [14, 17]. Amrein and coauthors [15], however, could not find a sex-specific difference after adjustment for age, bone mineral content, physical activity, body mass index, and renal function. Seasonal changes, with higher levels during wintertime, have also been described [19]. Since osteocytes are the main mechanosensors in bone, it is not surprising that sclerostin expression depends on mechanical loading. In an experimental setting, mechanical stimulation of bone (ulna) reduced the expression of sclerostin [20] and *SOST*−/− mice have been shown to be resistant to the bone loss caused by mechanical unloading [21]. In humans, study results differ a little bit. An exercise program lasting 12 months (resistance or jump training) led to decreases in serum sclerostin levels [22], but except for our investigation on ultradistance runners [23], no other trial detected a decrease of sclerostin following an acute exercise bout. The increase of inflammatory cytokines has been shown to be associated with the transient increase of serum sclerostin following a single workout [24]. Immobilized stroke patients had higher sclerostin levels than controls [25].

Considering the low or even unmeasurable serum levels of sclerostin in Van Buchem disease and sclerosteosis, the thought that high sclerostin levels lead to loss of bone mass and bone strength with an increased fracture risk is obvious. Therefore, several studies [16, 17, 26–29] evaluated the association between serum sclerostin level on one hand and bone mineral density (BMD), bone turnover markers (BTMs), and risk of fracture on the other hand (Table 1). Although low BMD is expected in subjects with high sclerostin levels, all studies so far have found a positive association between serum sclerostin and BMD. A possible explanation is that more bone means a higher number of osteocytes able to secrete sclerostin. All investigations found a negative correlation with BTMs. The association with fragility fracture risk is less clear. Some studies found a positive association, some did not. A reason for this discrepancy may be the method (different assays) of evaluating serum sclerostin levels. According to Arasu et al. [26] and Ardawi et al. [16], fracture risk is amplified in subjects with high serum sclerostin levels and low BMD. According to a very recent study, the bone protein content of the Wnt antagonists sclerostin and Dkk1 was positively correlated with bone mass and bone strength in postmenopausal women with previous fragility fracture [30].

**Table 1:** Association of the serum sclerostin level with bone mineral density (BMD), bone turnover markers (BTMs), and fracture risk

Population	Association with BMD	Association with BTM	Association with fracture	Reference
Older women	Positive	?	Positive	[26]
Postmenopausal women	No association except total body BMD	Negative	Positive	[16]
Postmenopausal women	Positive	Negative	No association	[27]
Older men	Positive	Negative	Negative	[28]
Institutionalized elderly women	Positive (SOS calcaneus)	Negative	No linear association	[29]
Elderly subjects	Positive (SOS calcaneus)	Negative	?	[17]

*SOS* speed of sound

## Preclinical studies and animal models

The knowledge that humans with genetic deficiencies of sclerostin have high bone mass induced in vitro and experimental investigations with the aim of developing anti-osteoporotic medication. Sclerostin knockout mice—imitating genetic deficiency of sclerostin—showed increases in bone formation, bone mass, and bone strength [31]. An overview of the effects of sclerostin deletion and overexpression on bone mass and bone strength in mice is given by Ke and coauthors [4]. Li et al. [32] mimicked postmenopausal osteoporosis and treated ovariectomized rats with a sclerostin-neutralizing antibody for 5 weeks. This procedure led to augmentation of trabecular, periosteal, endocortical, and intracortical bone formation. Treatment duration of 6 months corroborated the increases in bone mass and bone strength in ovariectomized rats, with more than 80% reductions in eroded surfaces [33, 34]. In line with these investigations is a study which showed increases of BMD and improved bone architecture in aged male rats after 5 weeks of subcutaneous administration of the sclerostin antibody compared with placebo treatment [35]. Androgen-deficient rats that received a sclerostin antibody subcutaneously starting 3 months after orchiectomy experienced an increase in bone strength (gain in bone mineral content accompanied with maintenance of bone quality) after 6 weeks [36]. A different study group corroborated these results: In adult female rats, 4 weeks of subcutaneous injection of a monoclonal sclerostin antibody led to increased bone formation and decreased bone resorption in trabecular bone [37]. Previous antiresorptive treatment with alendronate did not influence the anabolic effect of sclerostin antibody treatment in a negative way [38]. Transition from the sclerostin antibody application to vehicle application resulted in BMD loss; however, the transition to an antiresorptive agent after the cessation of sclerostin antibody treatment maintained the positive effect on BMD [39]. According to a colitis model, the treatment with a sclerostin antibody seems to counteract the accelerated bone loss associated with chronic inflammation [40].

The application of two monthly injections of a sclerostin neutralizing monoclonal antibody was evaluated in adolescent female cynomolgus monkeys. The effect of the antibody was dose dependent and BMD increases were up to 29% higher in treated (2 months) than untreated animals [41]. In ovariectomized cynomolgus monkeys, a one-year romosozumab treatment resulted in improvements in BMD as well as bone strength and maintained bone quality [42].

Fracture healing has been shown to be accelerated in *SOST* knockout mice as well as after sclerostin antibody treatment in wildtype rats [43, 44].

## Romosozumab

### Clinical studies

The first human study investigating romosozumab—a humanized monoclonal antibody directed against the osteocyte-derived glycoprotein sclerostin—was a single-dose investigation of 72 healthy subjects. Men and postmenopausal women received different doses of AMG 785 (former name of romosozumab) subcutaneously or intravenously. The substance was well tolerated and bone formation increased, whereas bone resorption decreased in a dose-dependent manner, leading to increases in BMD (lumbar spine +5.3%, total hip +2.8%) by day 85 [45]. A 3-month multiple dose investigation evaluated the effect of subcutaneous injections (1 or 2 mg/kg every 2 weeks of 2 or 3 mg/kg every 4 weeks) of romosozumab in 32 osteopenic postmenopausal women and 16 osteopenic men [46]. Depending on the exposure of romosozumab, the bone formation marker procollagen type 1 N-terminal propeptide (P1NP) transiently increased by 66–147% and the bone resorption marker C‑terminal telopeptide of type 1 collagen (CTX) decreased by 15–50%, leading to a BMD increase of the lumbar spine of 4–7%. A high-resolution quantitative computed tomography (HRpQCT) analysis of 48 subjects revealed a 9.5% augmentation of trabecular BMD induced by 3 months of romosozumab therapy [47].

A phase 2 study including 419 postmenopausal women investigated five different dosing regimens of romosozumab (70, 140, 210 mg per month, 140 or 210 mg every 3 months, or placebo injections). Additionally, patients of open-label study arms were on alendronate or teriparatide treatment [48]. After 12 months, lumbar spine BMD, which was the primary endpoint, showed an increase at all dose levels—11.3% with the 210 mg per month dose. That was significantly greater than the 4.1% increase with alendronate and the 7.1% increase with teriparatide. P1NP values peaked after 4 weeks; thereafter, the bone formation marker decreased to or even below baseline levels. ß‑CTX decreased within a week after the first romosozumab application and remained below baseline values during the whole study period. Analysis of a subset of the patients who had undergone QCT assessment revealed higher cortical vertebral volumetric BMD, higher trabecular hip volumetric BMD, and larger cortical bone mineral content gains with romosozumab compared with teriparatide at the spine and the hip [49]. Another substudy of this international phase II study evaluated bone strength gains using finite element analysis: Vertebral strength increased more for romosozumab compared with teriparatide (27.3% versus 18.5%) and placebo (27.3% versus −3.9%); femoral strength increased by 3.6% in the romosozumab group whereas it decreased by 0.7% in the teriparatide group and by 0.1% in the placebo group [50]. An extension of the international phase II study [48] was recently published [51]. Women in the romosozumab and placebo groups continued their treatment for an additional 12 months, the alendronate group transitioned to 140 mg romosozumab each month, and the teriparatide group was no longer part of the study. After 24 months, women were rerandomized to 60 mg denosumab or placebo every 6 months for another 12 months. Continuing romosozumab application for a second year led to further increases in BMD. However, these gains were a lot smaller than during the first year—at the lumbar spine 11.3% by month 12 and 15.1% by month 24, at total hip 4.1% by month 12 and 5.4% by month 24. Further increases in BMD could be observed in participants who transitioned from romosozumab 210 mg for 24 months to another 12 months of denosumab therapy. In subjects who received placebo after romosozumab treatment, BMD values decreased. Contrary to the lumbar spine and hip region, BMD at the 1/3 radius deceased modestly from baseline till 24 months while receiving romosozumab. Subjects who had been treated with an oral bisphosphonate previously showed only slightly lower BMD increases than treatment-naïve subjects. Another phase II study lasting 12 months performed in Japan which included 252 postmenopausal osteoporotic women showed similar results concerning BMD gains and the course of bone turnover markers [52].

**Fig. 1:**
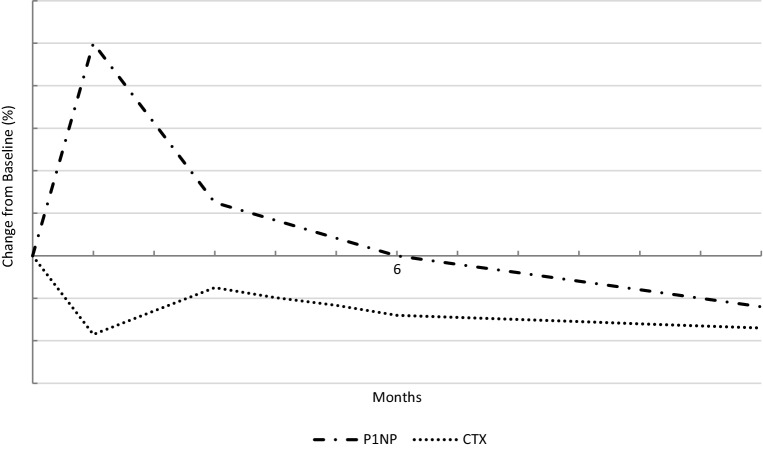
Schematic representation of bone turnover marker changes, the bone formation marker procollagen type 1 N-terminal propeptide (*P1NP*) and the bone resorption marker C‑terminal telopeptide of type 1 collagen (*CTX*), during a one-year application of romosozumab; adapted from [54, 57, 58]

The treatment effects of romosozumab have also been compared with the treatment effects of teriparatide in a randomized open-label phase III study [53]. Four hundred thirty-six postmenopausal women who had been taking alendronate for at least 3 years were randomized to receive romosozumab (210 mg/month) or teriparatide (20 µg/day). At the 12-month appointment, the mean change in hip BMD was significantly higher in the former group (+2.6% versus −0.6%). Integral volumetric bone mineral content was increased with romosozumab but unchanged with teriparatide, and finite element analysis also revealed greater gains in hip strength with romosozumab. The Fracture Study in Postmenopausal Women with Osteoporosis (FRAME) [54] is a double-blind study of 7180 postmenopausal osteoporotic women randomized to monthly subcutaneous injections of 210 mg romosozumab or placebo. After 12 months, both treatment groups received subcutaneous injections of 60 mg denosumab every 6 months for an additional 12 months. Within the first study year, the incidence of new vertebral fractures was reduced by 73% and the incidence of clinical fractures by 36% in the romosozumab group. At 24 months, vertebral fracture risk was 75% lower in women who started with romosozumab and switched to denosumab. Non-vertebral fracture risk only showed a group-specific difference, favoring romosozumab treatment (hazard ratio 0.58) in a post hoc analysis after excluding Latin American study participants because in that region, the risk of non-vertebral fractures was much lower than at all other study sites [55]. Another endpoint of the FRAME study [54] was BMD gains, which were higher in FRAME participants who received romosozumab for 12 months and denosumab for another 12 months, and approximated after 2 years of therapy the BMD gains after 7 years of continuous denosumab application [56]. Another phase III study, the Active-Controlled Fracture Study in Postmenopausal Women with Osteoporosis at High Risk (ARCH) [57], which enrolled 4093 postmenopausal women with high fracture risk (99% had a history of fragility fracture), compared 12 months of monthly subcutaneous romosozumab (210 mg) with weekly oral alendronate (70 mg). Afterwards, 12 months of alendronate therapy were added for all patients. After 12 months, vertebral fracture risk was 37%, clinical fracture risk 28%, and non-vertebral fracture risk 26% lower with romosozumab. After 24 months, vertebral fracture risk was 48% and clinical fracture risk 27% lower in the romosozumab-to-alendronate group than in the alendronate-to-alendronate group. Between-group differences of non-vertebral fracture risk (19%) and hip fracture risk (38%) did not reach statistical significance. The most recent phase III study, the BRIDGE study, included 245 men with a history of fragility fractures [58]. Concerning BMD as well as bone turnover markers, results of this placebo-controlled double-blind study were like those in postmenopausal women. BTM changes induced by romosozumab therapy are schematically presented in Fig. 1.

### Safety

During the development of a new medication, safety concerns are immanent. In a phase I study, one patient developed hepatitis, but increases in transaminase values have not been observed thereafter. In the phase II study published by McClung et al. [48], mild transient asymptomatic reductions in serum calcium were observed. Despite daily calcium and vitamin D supplementation, serum calcium levels decreased by 2% after 1 month of romosozumab therapy in the FRAME study [54]. One subject in the FRAME study and one subject in the ARCH study developed asymptomatic hypocalcemia. Injection site reactions varied between 4.4 and 5.5% for romosozumab application. Placebo-associated injection site reactions varied between 2.9 and 3.7%. In the FRAME study, one subject of the romosozumab group developed osteonecrosis of the jaw and one subject suffered from an atypical femoral fracture. The number of serious cardiovascular adverse events was imbalanced in the ARCH and the BRIGE trials (Table 2). In 15 to 20% of the patients, anti-romosozumab antibodies were detected during the first year of therapy but this did not have any effect on safety or efficacy. Since the Wnt signaling pathway is essential for cellular proliferation in several tissues, concerns were raised that malignancy might be induced by antisclerostin therapy [59]. According to a rat lifetime pharmacology study, however, there are no indications that romosozumab administration poses a carcinogenic risk to humans [60].

**Table 2:** Relevant side effects which occurred in phase III studies

	FRAME [54]	ARCH [57]	BRIDGE [58]
Duration	12 months	12 months	12 months
Patients	Women, 55–90 years	Women, 55–90 years	Men, 55–90 years
Design	Romosozumab (*n* = 3589) vs. placebo (*n* = 3591)	Romosozumab (*n* = 2046) vs. alendronate (*n* = 2047)	Romosozumab (*n* = 163) vs. placebo (*n* = 82)
Geographic region	Central Europe, Eastern Europe, Latin America, Western Europe, Australia. New Zealand, Asia-Pacific, North America	Central Europe, Eastern Europe, Middle East, Latin America, Western Europe, Australia. New Zealand, Asia-Pacific, South Africa, North America	Europe, North America, Latin America, Japan
Injection site reaction	187 (5.2) vs. 104 (2.9)	90 (4.4) vs. 53 (2.6)	9 (5.5) vs. 3 (3.7)
Hypocalcemia	1 (<0.1) vs. 0	1 (<0.1) vs. 1 (<0.1)	0 vs. 0
ONJ	1 (<0.1) vs. 0	0 vs. 0	0 vs. 0
Atypical femoral fracture	1 (<0.1) vs. 0	0 vs. 0	0 vs. 0
Serious cardiovascular event	44 (1.2) vs. 41 (1.1)	50 (2.5) vs. 38 (1.9)	8 (4.9) vs. 2 (2.5)

*ONJ* osteonecrosis of the jaw

To be on the safe side, McClung recommends refraining from romosozumab treatment in patients with hypocalcemia or vitamin D deficiency as well as in patients with the existence or the risk of skeletal metastases or other high bone remodeling conditions [61].

## Blosozumab

### Clinical studies

The safety and tolerability of blosozumab, another antibody against sclerostin, was studied in a randomized, placebo-controlled trial using single or multiple escalating doses for 8 weeks [62]. Blosozumab, which was given subcutaneously or intravenously to postmenopausal women, was well tolerated; bone formation markers increased and bone resorption decreased. An increase in BMD at the lumbar spine (+3.4% to +7.7%) could be observed after 85 days.

A phase II study included 120 postmenopausal women with low BMD [63]. The randomized, double-blind, placebo-controlled trial investigated different doses of blosozumab given subcutaneously—up to 270 mg every 2 weeks. The peak of the P1NP increase was reached 4 weeks after the first blosozumab application. CTX deceased and remained reduced through the whole study. According to follow-up after treatment cessation, BMD gradually decreases. However, lumbar spine and total hip BMD were still higher than initially placebo-treated women after 1 year [64].

### Safety

In the phase II study [63], injection site reactions were more frequent in the blosozumab than in the placebo group (up to 40% vs. 10%). Mild transient asymptomatic reductions in serum calcium were also observed in the blosozumab group. Serious adverse events were not related to the medication. Thirty-five percent of the subjects developed anti-drug antibodies. Because of the injection site reactions in the study performed by Recker et al. [63], Eli Lilly developed alternate formulations. In the second phase I study, however, injection site reactions still occurred. According to a statement of the company, “Lilly was unable to identify a tolerable formulation of blosozumab to move into phase II studies.”

## Further sclerostin antibodies

Novartis also developed a sclerostin antibody, BPS804, also called setrusumab, which has been sold in the meantime to the Mereo Biopharma Group PLC. Searching in PubMed, no publications of BPS804 and postmenopausal osteoporosis are identified. Only patients suffering from hypophosphatasia and osteogenesis imperfecta have been treated with BPS804 so far [65, 66].

## Approval of Evenity (romosozumab)

The registered trade name of romosozumab is Evenity. The first country to approve Evenity was Japan. There, in South Korea, and Australia Evenity is approved for the treatment of osteoporosis for women and men at a high risk of fracture. In Canada, osteoporotic postmenopausal women at high risk of fracture may be treated with Evenity. Since April 2019, this is also possible in the US, because at that timepoint, the Food and Drug Administration (FDA) gave the green light. However, according to the prescribing information [67] “Evenity may increase the risk of myocardial infarction, stroke and cardiovascular death. Evenity should not be initiated in patients who have had a myocardial infarction or stroke within the preceding year. Consider whether the benefits outweigh the risks in patients with other cardiovascular risk factors. If a patient experiences a myocardial infarction or stroke during therapy, Evenity should be discontinued.” In Europe, approval of Evenity was granted by 2019-12-12. The indication is “Treatment of severe osteoporosis in postmenopausal women at high risk of fracture” [68].

## Conclusion

Studies of patients with rare bone diseases and the understanding of the Wnt signaling pathway in bone metabolism identified the negative regulator of bone mass sclerostin as a potential target for the treatment of osteoporosis. Serum sclerostin levels are supposed to give a good impression of sclerostin levels in bone. However, circulating sclerostin is not currently evaluated in clinical routine, but rather only for scientific purposes. Two different antibodies against sclerostin have been investigated as potential treatment options for postmenopausal women. It has been proven that the sclerostin-binding monoclonal antibody romosozumab has advantageous effects on both aspects of bone volume regulation—it increases bone formation and reduces bone resorption. Within 1 year, BMD increases of more than 10% at the lumbar spine and as high as 7% in the hip region were observed. Vertebral as well as non-vertebral fractures were significantly reduced. Since the anabolic effect gradually gets lost after cessation of treatment, patients should go on with an antiresorptive treatment afterwards. At present, romosozumab is in clinical use in four countries. In Europe, we are still waiting for the final decision of the European Medicines Agency (EMA).
